# Climate Change and Herbivores: Forty Years in a Bunchgrass Prairie

**DOI:** 10.3390/ani14182647

**Published:** 2024-09-12

**Authors:** Gary E. Belovsky, Jennifer B. Slade

**Affiliations:** Department of Biological Sciences, University of Notre Dame, Notre Dame, IN 46556, USA; belovsky.2@nd.edu

**Keywords:** herbivore abundance, primary production, climate change, bunchgrass prairie

## Abstract

**Simple Summary:**

The wild terrestrial herbivore community (primarily insects and mammals) at a location should be affected by vegetation changes with anthropogenic climate change. Expected warmer and drier conditions should lead to a lower quantity and nutritional quality of plants. For 40 years, we studied the climate, the vegetation, and the assemblage of native herbivores (grasshoppers, microtine rodents, pronghorn, whitetail, mule deer, bighorn sheep, elk, and bison) in a grassland (National Bison Range, Charlo, MT, USA). Contrary to climate change expectations, forage quantity and quality increased, with grass abundance increasing more than broad-leaf plants decreased. This increased herbivory by nearly 20%, with grass-feeding herbivore abundance increasing (some grasshoppers, microtine rodents, bighorn sheep, elk, bison), while broad-leaf-feeding herbivores decreased (some grasshoppers, pronghorn, whitetail, and mule deer). Rather than studying the responses of single herbivore species, the assemblage needs to be studied, as some species may increase in abundance, and others may decrease. Historically, this assemblage of herbivores had grasshoppers consuming nearly 50% more vegetation than the mammals, but with climate change, grasshopper herbivory relative to mammals declined nearly 70%. Therefore, ecosystem dynamics have been altered by climate change over a short period, which has management consequences as desirable (e.g., endangered and harvested species) and undesirable species (e.g., pests) may differentially be affected.

**Abstract:**

Wild herbivore responses to anthropogenic climate change are often projected to be habitat and geographic range shifts as warmer conditions reduce the quantity and nutritional quality of forage plants, which makes species presence/absence a focus. Since 1978, herbivore abundances at the National Bison Range, MT, USA, were measured for grasshoppers (catch-effort), microtine rodents (runway density), and ungulates (drives and round-ups), along with climate and vegetation quantity (biomass) and quality (nitrogen content and chemical solubility related to digestibility). Counter to expectation with warming and drying, forage biomass increased as grass biomass increased more than dicot biomass decreased, and forage quality (solubility) increased. Consequently, herbivores that consume a grass diet (>25% grass: certain grasshoppers, microtines, bighorn sheep, elk, bison) increased in abundance, while herbivores consuming less grass declined (certain grasshoppers, pronghorn, whitetail, and mule deer). The result is an 18% increase in herbivore abundance and herbivory, counter to climate change expectations. Historically, grasshoppers consumed 46% more vegetation than mammals; now, they consume only 14% more, as grasshoppers did not increase as expected with climate change. Therefore, herbivores respond rapidly to climate-induced vegetation changes, and this is not a simple loss/addition of species, but changing trophic dynamics, which requires more knowledge of ecosystem dynamics.

## 1. Introduction

The effects of anthropogenic climate change on animals have focused on shifts in range and habitat availability within and across regions (e.g., [1,2,3,4,5,6,7,8,9,10]). This focus fosters assessments based on species presence/absence and habitat data, which are relatively easy measurements to obtain. However, changes in population density and demography at local scales may be a more common impact of climate change, especially in the short term, and this will affect ecological communities, especially trophic dynamics (e.g., [1,4,6,11,12,13,14,15,16,17,18,19]). These population and community responses are more difficult to obtain, requiring detailed monitoring and field experiments.

Because semi-arid and arid grasslands account for up to 40–50% of Earth’s land mass and are critical for livestock production, how climate change may adversely impact livestock as vegetation responds (forage quantity and quality) to climate change has been a focus of study [20,21,22,23]. Climate change (warmer and drier conditions) is proposed to decrease forage abundance and quality (e.g., lower nitrogen content) for livestock. Grasslands are also important to wildlife and their conservation, as well as to insect pests and their control [24,25]. Herbivorous wildlife [21] and insects [26,27,28,29] should also respond to the reduced forage abundance and quality proposed with climate changes. However, in addition to forage changes, wildlife and insect pests may be more subject to changing thermal conditions than livestock, which are shielded by humans, as thermal conditions can modify ecological dynamics (feeding, competition, and predation: [18,20,30,31]).

We have documented climate changes over the past 105 years in an intermountain bunchgrass prairie at the Bison Range (BR) in MT, USA [32]. Climate changes have dramatically impacted vegetation and ecosystem processes over the past 40 years. Annual aboveground net primary production (ANPP) surprisingly increased as June became cooler and wetter, even in the face of annual warming/drying, but this production rapidly senesced as summer drought intensified, and the production has become dominated by grasses. These new ecosystem dynamics should affect herbivores. We examine here how dominant invertebrate (grasshoppers: Orthoptera, Acrididae) and mammalian (rodent and ungulate) herbivores responded to these vegetation changes over the past 40 years.

## 2. Materials and Methods

### 2.1. Study Site

The National Bison Range (U.S.F.W.S.) in Montana, now the Bison Range (BR) since transfer to the Confederated Salish and Kootenai Tribe in 2020, is one of the largest remaining tracts (~9000 ha) of bunchgrass prairie, an endangered ecosystem (<1% remaining) that once covered intermountain valleys from the Rockies west to the Cascades and from Southern Alberta/British Columbia south to northern Utah/Nevada [33]. BR was never farmed and has been protected since 1908 as a refuge for bison (*Bison bison*). Prior to this, it was a cattle/bison ranch for less than 15 years. Elevation varies from ~800 to 1400 m. 

We [32] reported on BR vegetation changes over the past 40 years. The ANPP averaged 191.5 (±8.6 SE) g/m^2^ (range: 100.9–316.4 g/m^2^), with grass accounting for 79.2% (±2.2 SE) of the ANPP (range: 41–97%) and summer drought reducing live-plant biomass by 59.2% (±2.5 S.E.) (range: 30.1–85.6%). Over the past 40 years, we found that the BR ANPP increased by 110%, as the period of greatest production (late May–June) became wetter and cooler. Grass production increased by 251%, while dicot production declined by 65%, which increased grass relative abundance by 54%. Summer temperatures increased 12.5%, which increased plant senescence by 119%. BR has an abundant array of wild herbivores (grasshoppers, microtine rodents, and ungulates). 

Grasshoppers (Orthoptera, Acrididae) are historically the dominant bunchgrass herbivores [34,35,36], and are generally more abundant than in the Great Plains [34]. For example, historical plagues of the now-extinct Rocky Mountain Plague Locust (*Melanoplus spretus*) that devastated the Great Plains primarily emerged from bunchgrass regions and migrated east [37]. More than 25 grasshopper species occur at BR; all are univoltine and all but two overwinter as eggs. A late spring/early summer grasshopper assemblage composed of 5 species is never abundant (<<1 adult/m^2^). The summer assemblage is generally very abundant (>5 adults/m^2^), with one species, *Melanoplus sanguinipes*, dominating (50–90% of biomass).

Two microtine rodents (*Microtus pennsylvanicus*, *M. montanus*) and six ungulates (bison: *Bison bison*; elk: *Cervus elaphus*; bighorn sheep: *Ovis canadensis*; mule deer: *Odocoileus hemionus*; whitetail deer: *Odocoileus virginiana*; pronghorn: *Antilocapra americana*) are abundant. Ungulates historically may have been less abundant and consumed less vegetation than on the Great Plains [38,39,40,41,42]. While this is debatable, BR mammalian herbivores, especially ungulates, are abundant (~2.5 g/m^2^), approaching Great Plains historical levels (~5 g/m^2^: [43,44]).

Grasshopper biomass (~5 g/m^2^) is greater than mammalian herbivore biomass (~2.5 g/m^2^), but varies more among years. On average, grasshoppers cut (not necessarily consume) ~5 times more plant biomass per unit of body mass than mammals, which results in annual plant loss that is 1.25–1.67 times greater than that from mammals, even though grasshoppers are present for only 4–5 months per year. Herbivory at BR can approach 40% of plant ANPP [45].

### 2.2. Methods

Field Research—permission granted by National Bison Range, Charlo, Montana—https://www.bisonrange.org/, accessed on 10 September 2024.

#### 2.2.1. Plant Nutritional Traits

Two nutritional traits for herbivores were measured on grass and dicot samples collected by Belovsky and Slade [32], and not reported elsewhere. Plant N was measured by micro-Kjeldahl methods from 1994 to 2000 [46] and by elemental analyzer (^©^Costech) from 2001 to 2019 [47]. Results from the two methods for samples collected in 2000 were found to be comparable (r^2^ = 0.96, N = 6, *p* < 0.003: std. slope = 0.97, std. intercept = 0). In addition, plant solubility in HCl and pepsin [46] was measured on the same grass and dicot samples from 1985 to 2019 as an index of digestibility to herbivores [48]. Although the solubility can be correlated with actual digestibility for specific herbivores, a quantitative conversion is species-specific given digestive physiology and the selection of plant parts [49,50,51,52]. However, a simple measure of relative plant nutritional quality, like solubility, rather than more involved measures, was needed given that each year up to 960 plant samples needed to be evaluated (10 samples every 2 weeks/area × 12 weeks × 4 areas × 2: grass and dicot separated [32]). 

#### 2.2.2. Grasshoppers

For 1978–2019, density (#/m^2^) was measured in two ways at 8 sites (described in [32]):

- 1978–1985: A 100 m^2^ area was encircled with an insect screen (1.52 m high) at the time of peak adult density. Density was estimated by having two individuals using insect nets catch all individuals for 15 min and count any individuals that flew over the insect screen. These counts were performed 3–4 times in an area, and used in a “catch-effort” method [53]. The method uses regression of the sum of all grasshoppers caught in the area prior to a 15 min catch period as the independent variable and the number caught in the current 15 min catch period as the dependent variable. The regression line’s x-intercept estimates the numbers within the area with a 5–20% CV. Grasshoppers were preserved in 70% ETOH.
- 1986–2019: Density was estimated biweekly from early June to late Sept at each site using 24–40, 0.1 m^2^ rings [54]. Density estimate CVs were <10%. In addition, a sweep sample of 50 individuals for each biweekly census was preserved in 70% ETOH for species, life stage, and sex identification [55,56,57,58,59,60,61,62,63,64,65]. Using these data, additional metrics were computed for each site:
  - Average density is the mean of all non-zero censuses;
  - Peak density is the highest observed density among the censuses;
  - Maximum density is the sum of increases in density between consecutive censuses;
  - Days to 50% mortality is the time for peak density to decline by 50%, using interpolation between consecutive censuses;
  - Adult lifespan is the ordinal day of the Julian year when most individuals die due to freezing (~day 240) minus the ordinal day of the Julian year on which 50% of the population is an adult, using interpolation between consecutive censuses;
  - Hatching day is the ordinal day of the Julian year when (non-overwintering) grasshoppers are first observed.

#### 2.2.3. Microtines

For some years (1980–1987, 1990, 1992–2007, 2009, 2011–2019), an index of microtine abundance was obtained at 1–10 (mean = 4 ± 0.45 SE) random locations on the BR by counting the number of runways crossing a 50 m transect [66]. Runways are trails made by microtines as they forage and move between their burrows and tunnels [67].

#### 2.2.4. Ungulates

Ungulates were censused by BR staff (BR refuge unpubl. records). Elk (1978–2012, 2014–2015, 2017), bighorn (1978–2017), mule deer and whitetail (1978–2000, 2003–2005, 2010–2011) and pronghorn (1978–2011, 2014–2015) were counted by fall drive surveys over the entire refuge, and there was no regular management of these species. Bison (1978–2018) were counted in October by rounding-up essentially the entire population except a few bulls, and population numbers were managed by the sale of excess individuals. Bison numbers were also managed by moving them between large paddocks and by vaccination for livestock diseases. We only examined their numbers through 2007, because their numbers were managed at lower levels after this. Ungulate numbers were converted to biomass using values from Belovsky [49].

#### 2.2.5. Statistics

SYSTAT 13 was used to conduct analyses of the dependent variables of plant nutritional traits and herbivore measures that are continuous, normally distributed, and not overdispersed. These data were transformed if necessary to achieve normality and prevent overdispersion (variance/mean > 1); all proportions were arcsine square root-transformed. If the dependent variable was sampled over the entire BR (mammal data), then General Linear Models (GLMs) were employed where year was a categorical independent fixed variable to assess whether the dependent variable varied among years. If the dependent variable was sampled repeatedly at specific BR locations (sites) among years (plant and grasshopper data), then General Linear Mixed Models (GLMMs) were employed where year was a categorical independent fixed variable and site was a categorical random variable, as site was not replicated within a year.

To assess whether the above dependent variables exhibited trends over the years of our study, two methods were employed. First, if a single annual value was measured and the time series was uninterrupted (no missing years), nutritional and herbivore trends over time were analyzed using nonparametric trend analysis (Mann–Kendall rank correlation). Second, if values were measured for different sites or the time series was interrupted (missing years), nutritional or herbivore trends were analyzed using the above described GLM and GLMM analyses, where year since 1978 is a continuous integer fixed variable and site is a categorical random variable. Finally, percent change in values over our study (40 years) was computed as its trend by solving its linear regression in 1978 versus 2018.

Annual average herbivore population responses were examined in regard to food quantity and quality measures, plant senescence, and temperature as independent variables using multiple regression. This was performed using SYSTAT 13’s Best Subset regression option, where independent variables were only included if *p* < 0.15 and they decreased the Akaike Information Criteria value (AIC).

## 3. Results

The BR data for 1978–2019 are provided in Supplemental Table S1.

### 3.1. Plant Nutritional Traits

**Figure 1:**
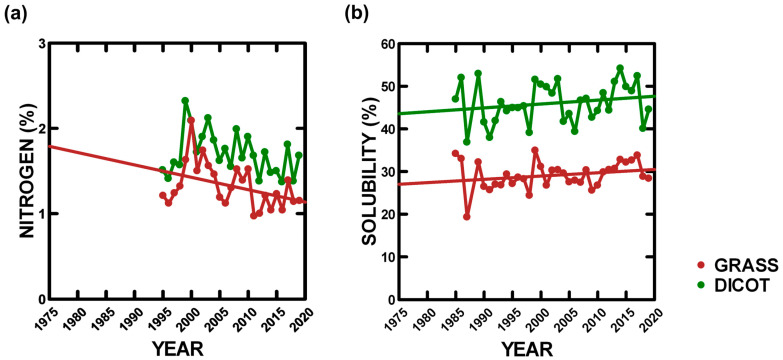
Annual grass and dicot nutritional characteristics averaged across all sites: (**a**) nitrogen and (**b**) solubility. Statistically significant linear trend lines are provided (Table 1).

Plant nitrogen content averaged over our study was 1.3% (±0.05 SE: among years: 0.97–2.09%) for grasses and 1.70% (±0.05 SE: among years: 1.37–2.32%) for dicots. Grass and dicot nitrogen content significantly varied among years, with grass nitrogen content significantly decreasing by 34.1% over our study (GLMM: Table 1, Figure 1b). Grass and dicot nitrogen content were positively correlated (r = 0.63, N = 94, *p* < 0.00001). Plant solubility in HCl and pepsin, an index of digestibility to herbivores, averaged over our study was 29.1% (±0.6 SE) (among years: 19.3–34.9%) for grasses and 46.0% (±0.8 SE) (among years: 36.9–54.2%) for dicots. Grass and dicot solubility significantly varied among years, with grass solubility increasing by 11.3% and dicot solubility by 8.3% over our study (GLMM: Table 1, Figure 1a). Grass and dicot solubility were positively correlated (r = 0.64, N = 117, *p* < 0.00001). 

Plant nitrogen content is positively correlated with solubility (r = 0.52, N = 192, *p* < 0.00001), even though, over time, solubility increased and nitrogen content decreased. This occurred because nitrogen content also decreased as ANPP increased over time [32]. Therefore, when ANPP was combined with plant nitrogen content to examine solubility, ANPP was positively correlated with solubility (*p* < 0.0003), and this improved the correlation (Table 1, r = 0.56).

### 3.2. Herbivores

Changes in herbivore abundances, species diversity, and species richness over our study were examined, as well as a number of additional grasshopper demographic and phenological characteristics.

#### 3.2.1. Grasshoppers

**Figure 2:**
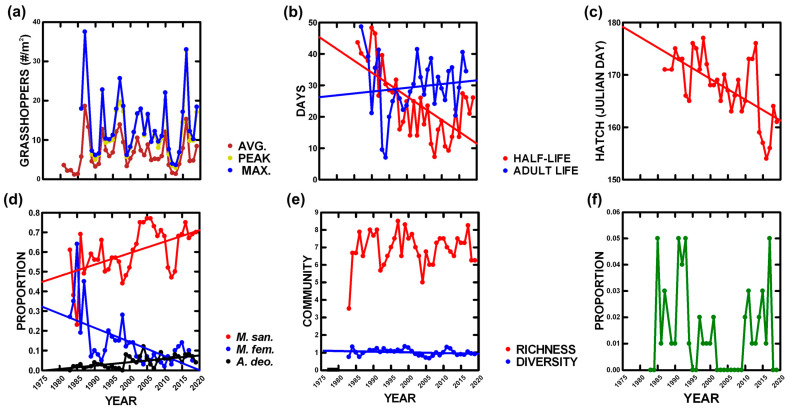
Annual grasshopper measurements averaged across sites with statistically significant linear trends (Table 2): (**a**) average, peak, and maximum density, (**b**) days to attain 50% mortality (half-life) and 50% adults (adult life) in the population, (**c**) hatching date, (**d**) relative abundance of three common species, (**e**) species richness and diversity, and (**f**) proportion of grasshoppers in the spring community.

Maximum, peak, and average densities varied among the years but did not change over our study (Table 2, Figure 2a). Several demographic traits were observed to change. First, the half-life of individuals in the populations (days) varied among the years, and decreased by 68.7% (increased mortality) over our study (Table 2, Figure 2b). Second, adult lifespan varied among the years and increased by 18.0% over our study (Table 2, Figure 2b). Finally, Julian day at hatching varied among the years and occurred earlier, decreasing by 8.9%, over our study (Table 2, Figure 2c).

Grasshopper species composition and diversity were examined. First, the proportion of grasshoppers representing a spring community (*Melanoplus confusus*, *M. oregonensis*, *Arphia conspersa*, *Chortophaga viridifasciata*) varied among the year but did not change over our study (Table 2, Figure 2f). Second, three summer community species (*Melanoplus sanguinipes, M. femur-rubrum*, *Ageneotettix deorum*) were abundant enough to follow; two varied among the years (*M. sanguinipes* and *M. femur-rubrum*), and all three changed over our study (*M. sanguinipes* increased by 50.5%, *A. deorum* increased by more than 3000%, and *M. femur-rubrum* decreased by 92.2%) (Table 2, Figure 2d). Finally, species richness did not vary among the years, and it did not change over our study (Table 2, Figure 2e), while Shannon diversity varied among the years and decreased by 13.5% over our study (Table 2, Figure 2e).

#### 3.2.2. Mammals

**Figure 3:**
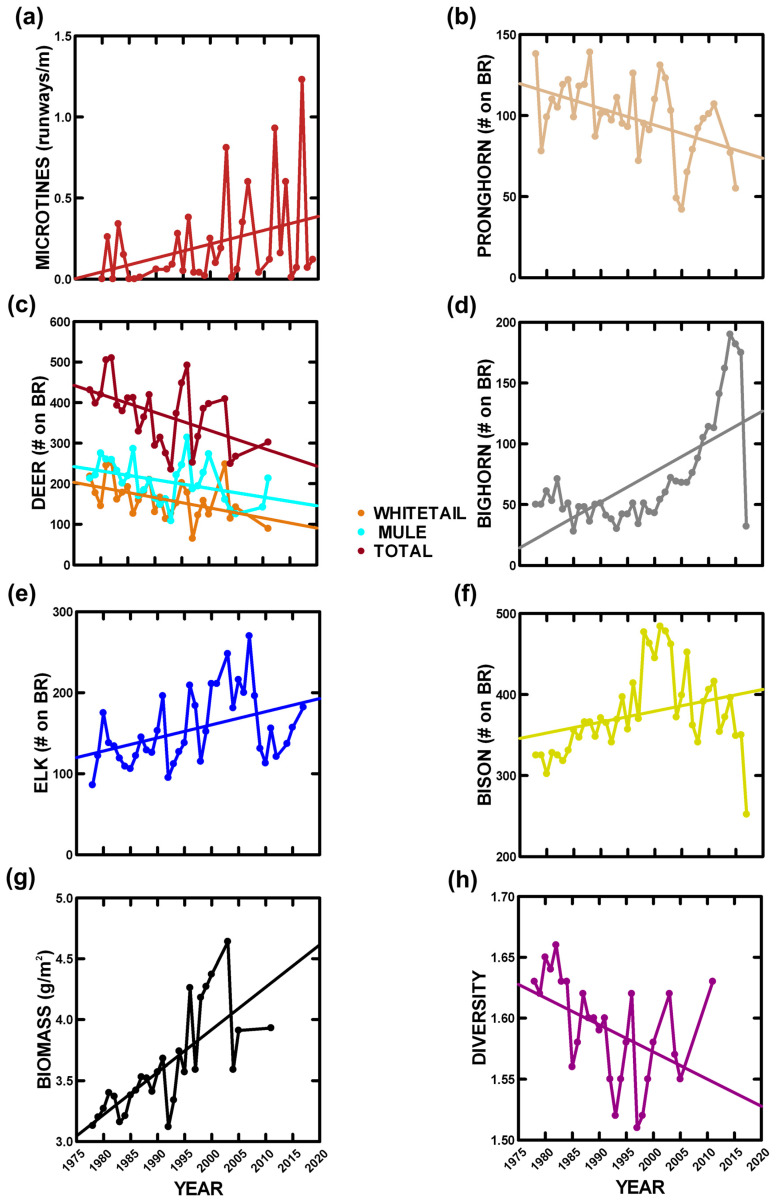
Annual mammalian herbivore measurements with statistically significant linear trends (Table 3): (**a**) microtine runway densities, (**b**) pronghorn numbers, (**c**) deer numbers, (**d**) bighorn sheep numbers, (**e**) elk numbers, (**f**) bison numbers, (**g**) ungulate biomass density, and (**h**) ungulate diversity.

Microtine runways, a density index, were highly variable among years (Figure 3a), as expected given a microtine tendency to cycle over 3 years, and increased by more than 700% over our study (Table 3), especially for peak abundance (*p* < 0.0002). Three ungulates (pronghorn, whitetail deer, mule deer) declined over our study (respectively, 34.8%, 50.6%, and 36.2%), while three (bighorn, elk, bison) increased over our study (respectively, 513.7%, 52.6%, and 15.4%) (Figure 3b–f, Table 3). Bison exhibited a weak response, due to intensive management, particularly due to reduced abundance by managers since 2007. If bison abundance prior to 2007 is examined, it increased by 46.6% (*p* < 0.00001). Ungulate biomass increased by 44.7% (Figure 3h, Table 3) and species diversity decreased by 5.8% (Figure 3g, Table 3) over our study.

## 4. Discussion

Our studies in this bunchgrass prairie have exhibited trends over the past 40 years that document vegetation responses to climate change [32], and we present here how the abundances of the main herbivores (Orthoptera, microtine rodents, and ungulates) have also changed. These herbivore responses could be directly due to abiotic (e.g., temperature) changes or responses due to biotic changes (e.g., vegetation affecting food availability), and we discuss these possibilities.

### 4.1. Climate Change, Vegetation, and Its Nutritional Traits

Climate change is proposed to reduce grassland plant production and nutritional quality, which can negatively impact herbivores [20,21,22,23], but this is not supported by our 40-year study.

- Plant production (ANPP) increased by 110% over our study, counter to climate change claims, due to increased precipitation and cooler temperatures in spring that foster growth, even though the annual climate has become drier and warmer [32]. ANPP increases were due to grass biomass increasing by 251%, as dicot biomass declined by 65%, and grasses benefit from warmer and drier summers [32]. Finally, this was not due to BR management increasing grass ANPP for bison to consume, because the vegetation was not managed (except some weed control) and ANPP was measured without herbivory [32].
- The nitrogen content of plant tissue (%) declined over time (Figure 1a, Table 1) in accord with climate change projections (grass by 34.1% and dicots by 19.6%). This occurred as soil nitrogen, a limiting nutrient for plant growth, increased with warming summers, but ANPP increased even more, which leaves less nitrogen for allocation to plant tissue [32].
- Plant solubility, an index of digestibility to herbivores, increased over time (grass by 11.3% and dicots by 8.3%), opposite of climate change projections (Figure 1b, Table 1). Plant solubility and nitrogen content are positively correlated, which is observed even though nitrogen content declined over time. This occurred because solubility also increased as ANPP increased (Table 1) over time [32], as experimentally demonstrated [68], and nitrogen content decreased as ANPP increased. Finally, solubility declined more rapidly within a year as plant senescence (vegetation “browning”) accelerated with increased summer warming and drying [32].
- Food availability indices can be computed as products of grass and dicot ANPP with their respective solubilities (e.g., grass food availability index = grass ANPP X grass solubility), which weights vegetation quantity by an index of nutritional quality. Grass food availability indices increased by more than 700% over our study (r = 0.84, N = 34, *p* < 0.00001), while dicot food availability indices decreased by 63.1% (r = 0.47, N = 34, *p* < 0.005), even though dicot solubility increased. Indices for total food (grass food availability index plus dicot food availability index) increased over our study by 148.8% (r = 0.71, N = 34, *p* < 0.00001). These indices are not an absolute measure of food availability for each herbivore species, as each may select different plant species and parts based on nutritional quality, but they do incorporate seasonal effects, as the solubility values are averaged over the year. However, the indices are a useful expedient for examining trends in food availability at a large temporal/spatial scale, as we address here. Therefore, one herbivore species may be a more selective feeder than another, but over time, both have more food available as the index increases.

Therefore, the grassland climate change projections that overall food quality and quantity for herbivores will decline [21,22,23] are not supported. Rather, we observe an overall increase in food quality and quantity, as grass quality and quantity increase, while dicot quality increases and quantity decreases, which leads to an overall increase in herbivore food through grasses at the expense of dicots.

### 4.2. Herbivore Responses to Climate Change

#### 4.2.1. Grasshoppers

Demography is driven by temperature and food factors (Table 4).

- Hatching date as expected occurred earlier as June warms.
- The proportion of grasshoppers representing a spring community increased, as expected with warmer springs (June) and more food remaining from the previous year (% “green” vegetation remaining) before new growth occurs.
- Half-life decreased (more rapid mortality) with the stress of a hot summer and lower nutritional foods for nymphal grasshoppers (greater grass relative abundance), but increased (slower mortality) with warmer springs (reduced cold after hatching).
- Adult lifespan increased with warmer June temperatures (faster maturation) and greater grass relative abundance (grass as food senesces slower, so that it remains “green” longer, which maintains nutritional quality).

Relative abundances of different grasshopper species varied with their feeding behavior [49,69,70]. *M. sanguinipes*, which consumes more grass and more senescent vegetation than *M. femur-rubrum*, increased in relative abundance as percent grass abundance increased, the reverse of *M. femur-rubrum* (Table 4). *A. deorum*, which also primarily consumes grasses and even more readily consumes senescent vegetation, increased in relative abundance as percent grass abundance increased, grass solubility increased, and June temperature increased (Table 4). 

Species richness declined as grass relative abundance increased and increased as dicot solubility increased (Table 4). Species diversity declined with warmer springs and with greater grass relative abundance (Table 4). These results suggest that richness and diversity increase with the addition of dicot-feeding species.

#### 4.2.2. Mammals

Like grasshoppers, mammalian herbivores that tend to consume more grass increased in abundance (microtines, bighorn, elk, bison), while those that tend to consume more dicot decreased in abundance (pronghorn, whitetail, mule deer) [49]. These changes are consistent with the increased grass and decreased dicot ANPP. Finally, this was not due to BR management increasing grass ANPP for bison to consume, as indicated above.

#### 4.2.3. Herbivore Community Changes

From an ecological perspective [71], population numbers of each herbivore species, their combined abundances, and their relative abundances (species diversity) are the main concern given climate change. Climate change projections for grasslands [21,22,23] suggest that overall herbivore abundances should decline because food availability should decline. However, based on observed BR herbivore diets [49], we observed that herbivores with a diet of more than 25% grass (*M. sanguinipes*, *A. deorum*, microtines, bighorn, elk, bison) increased in absolute and relative abundances, while those eating less grass (*M. femur-rubrum*, pronghorn, whitetail, mule deer) decreased; nonetheless, overall herbivore abundance increased. These differential responses are consistent with the vegetation changes we observed: the grass food availability index increased and dicot food availability index decreased.

Even though the overall grasshopper absolute abundance did not change, the relative abundance of grasshoppers that eat more grass increased. While grasshoppers were previously the dominant BR herbivore, they have diminished in importance compared to mammals, as mammalian herbivores increased. We might expect mammalian herbivores to increase more than grasshoppers, as mammalian herbivores are much more likely to consume “brown” senesced plants than are grasshoppers, as senesced plants increased with warmer and drier summers (see above).

The above absolute abundance trends suggest that each BR herbivore’s total population number or density at time t (N_t_) should be a function of its main food resource (grass ANPP, dicot ANPP, grass solubility, dicot solubility, grass food availability index = grass ANPP X grass solubility, dicot food availability index = dicot ANPP X dicot solubility) and perhaps with other factors (e.g., temperature, summer senescence of plants, etc.). For each herbivore, we fit a density-dependent population model for food limitation [72] using regression:

∆N = N_t_ − N_t−1_ = α N_t−1_(food_t−1_/N_t−1_ − β), (1)

where N_t−1_ is the previous year’s population or density, food_t−1_ is the previous year’s index of food availability (both grass and dicot as independent variables), β is the herbivore’s per capita food requirement for maintenance and replacement reproduction, and α is offspring produced per unit of food intake above β. This simplifies to

N_t_ = α food_t−1_ + (1 − αβ) N_t−1_, (2)

which is a linear function where α and β can be treated as regression constants. Plant senescence (fraction of ANPP still “green” in September) and summer temperature were also included in regressions, as additional climate change effects. Regression models for each herbivore are provided in Table 5, with the model producing the lowest AIC value highlighted.

The average grasshopper density was examined, as it was highly correlated with peak and maximum density (respectively, r = 0.95 and r = 0.78, N = 125, *p* < 0.000001). The current year’s average density was positively correlated with the prior year’s index of grass food availability and negatively with the current year’s summer temperature (Table 5). Therefore, average grasshopper density is a trade-off between food availability and, probably, the thermoregulatory effects of climate (temperature), as expected [73,74,75,76,77,78] and also observed in their demographic and phenological traits (Table 4). As grasshopper abundance is a composite of all species, this suggests that species consuming more dicots, as observed, are being disproportionally replaced by species consuming more grass, given the positive influence of the index of grass food availability on grasshopper abundance.

All mammalian species that decreased over our study (pronghorn, whitetail and mule deer) have the dicot food availability index in their regression (Table 5). All mammalian species that increased (microtines, bighorn, elk, bison) have the grass food availability index in their regression (Table 5). Bison are the only mammalian species affected by a factor other than a food availability index, as bison numbers increase in years when plant senescence is less.

Therefore, herbivore abundances always depend on grass or dicot food availability indices, which always provide much better regression fits than either grass or dicot ANPP. Only grasshoppers exhibit a direct effect of an abiotic factor, summer temperature, which is detrimental, even though we know that BR mammalian herbivores exhibit behavioral and feeding responses to thermoregulatory conditions [79]. We were also surprised with the absence of plant senescence appearing in the regressions for grasshoppers or mammals, except bison, as the dry brown vegetation due to summer drought has a lower nitrogen content and is less soluble, suggesting poorer nutritional value. Furthermore, we did not observe a general increase in grasshopper abundance [31] or a decline in mammalian herbivore abundance [20,22,23], as postulated for grasslands, with climate change. Rather, we observed an overall increase in herbivore abundance and a shift to herbivores that feed more on grass. Finally, the increase in mammalian herbivores and no change in grasshopper abundance means that grasshoppers are declining in their impact on the community from what was originally observed (see Section 2.1 [45]).

### 4.3. Implications for Vegetation–Herbivore Dynamics with Climate Change

As found for BR vegetation responses to climate change [32], searching for large-scale spatial patterns (e.g., Great Plains) based on changing average yearly climate observations (e.g., temperature and precipitation) may be misleading. Rather, responses need to focus on smaller scales (e.g., particular ecosystems) and seasonal climate changes (e.g., spring precipitation and temperature). Furthermore, BR herbivores reinforce this cautionary approach, as species responses to vegetation changes varied with their feeding behavior, with some decreasing in abundance and others increasing, while herbivores overall increased. Therefore, while studies that examine how individual species may respond to climate change are interesting (e.g., [80,81,82]), they do not address the responses of local ecosystems as we do and as others suggest [83,84].

BR vegetation changes over our 40-year study [32] have more than “rippled” through the herbivore trophic level. Consider that average grasshopper abundance did not change, microtine abundance increased by more than 700%, and ungulate biomass (g/m^2^) increased by 44.7%. Historically, herbivory in this ecosystem was dominated by grasshoppers, but now mammals are increasing in importance, given microtine rodents and grass-feeding ungulates. At the start of our study, grasshopper consumption was ~46% greater than that for mammals; now, it is only ~14% greater. Furthermore, overall herbivory increased by ~18% during our study, and this is due to mammals. Therefore, future changes in vegetation and herbivory may be very different from simple extrapolations of past to current conditions, requiring more detailed information.

As a consequence, wild herbivore conservation and management plans for climate change will require much more information on changes in herbivore behavior and the vegetation than often available. Furthermore, pooling data in meta-analyses (e.g., [29,84,85,86]) may obscure local effects brought about by climate change (e.g., [87]). Even examining data over time from a single location and trying to attribute trends to climate change without examining other underlying environmental changes (e.g., [88]) can be misleading [10,19]. This even applies to forecasting climate change impacts on domestic livestock production based on economics [88,89] and its greenhouse gas production [90,91].

## 5. Conclusions

Climate change over the past 40 years has affected bunchgrass prairie in expected and unexpected ways, changing the plant community [32], and we show that herbivores have responded rapidly to these vegetation changes. Grasshoppers, the dominant herbivore, declined in importance and mammals increased in importance, counter to climate change expectations [20,22,23,31]. Therefore, trophic dynamics (increased herbivory) rapidly responded; not through the loss or addition of species, but through modified abundances of the species already present.

Specific interactions (e.g., competition, predation, mutualisms, etc.) among species must be changing to modify herbivore abundances, but this information is not available. Interestingly, herbivore responses to changing food abundances indicate food limitation, not predator limitation as often assumed in trophic theory [92,93,94,95,96]. This could arise if predators cannot keep pace with herbivores as vegetation changes, but this cannot be the explanation as earlier BR studies [48,49,50,97,98,99,100] observed herbivores to be food-limited. Nonetheless, trophic dynamics appear to be more sensitive to climate change than often considered and to be changing in unexpected ways. Therefore, the knowledge needed to forecast the ecological effects of climate change may be deficient.

## Figures and Tables

**Table 1:** GLMM results for grass and dicot solubility and nitrogen content with year as an independent variable and site as a random variable. Annual differences (Among Years: year as a categorical variable) and trends over the years of our study (− = decline, 0 = no change, + = increase: year as a continuous variable) are examined. Trends over the years are also examined with ANPP included as a continuous variable. Finally, the relationship between solubility and nitrogen (with and without ANPP included) is presented. Significant (*p* < 0.05) results are in bold.

	Among Years			Over Years			
	df	F	*p*	Trend	df	F	*p*
SOLUBILITY −							
Grass ^1^	33,85	4.88	**0.00001**	+	1,117	10.78	**0.001**
Dicot ^1^	33,78	4.25	**0.00001**	+	1,110	8.50	**0.004**
vs. ANPP				0	1,239	0.33	0.57
NITROGEN −							
Grass ^1^	24,72	5.83	**0.00001**	−	1,95	12.59	**0.0006**
Dicot ^1^	24,66	3.68	**0.00001**	0	1,89	2.90	0.09
vs. ANPP				−	1,192	12.67	**0.0005**
SOLUBILTY vs. NITROGEN −							
Alone				+	1,190	69.86	**0.00001**
With ANPP				+,+	2,189	43.94	**0.00001** 0.0004

^1^ Arcsin sqrt transform.

**Table 2:** Mixed model GLMM results for grasshoppers among the years, and trend over the years of our study (− = decline, 0 = no change, + = increase) with site as a random variable. Significant (*p* < 0.05) results are in bold.

	Among Years			Over Years			
	df	F	*p*	Trend	df	F	*p*
DENSITY −							
Average ^1^	38,99	2.86	**0.0002**	0	1,136	0.69	0.41
Peak ^1^	32,87	2.30	**0.001**	0	1,118	0.05	0.83
Maximum ^1^	32,87	2.26	**0.002**	0	1,118	0.0002	0.99
DEMOGRAPHY							
Half-life ^2^	32,85	3.16	**0.0001**	−	1,116	27.90	**0.00001**
Adult lifespan ^2^	29,58	4.53	**0.00001**	+	1,86	4.92	**0.03**
PHENOLOGY							
Hatch date ^2^	31,54	5.53	**0.00001**	−	1,84	41.2	**0.00001**
Spring community ^3^	35,91	2.01	**0.004**	0	1,125	0.98	0.32
SPECIES							
*M. sanguinipes* ^3^	35,91	2.87	**0.00003**	+	1,125	22.86	**0.00001**
*M. femur-rubrum* ^3^	35,91	3.52	**0.00001**	−	1,125	35.14	**0.00001**
*A. deorum* ^3^	35,91	1.50	0.06	+	1,125	20.47	**0.00001**
Richness	35,91	1.29	0.17	0	1,125	0.49	0.48
Diversity	35,91	1.82	**0.01**	−	1,125	8.66	**0.004**

^1^ Ln transform; ^2^ Box–Cox transform; ^3^ Arcsin sqrt transform.

**Table 3:** Trend analyses (regression or Mann–Kendall) for mammalian herbivores over our study (− = decline, 0 = no change, + = increase). Significant (*p* < 0.05) results are in bold.

				Over Years	
	Trend	N	r	z	*p*
**Microtines −**	+	35	0.35		**0.04**
**Pronghorn −**	−	36	0.47		**0.004**
**Deer −**	−	27	0.50		**0.008**
**Bighorn sheep − ^1^**	+	39		4.14	**0.00002**
**Elk −**	+	37	0.42		**0.01**
**Bison − ^2^**	+	40		2.90	**0.002**
**Ungulate biomass −**	+	27	0.74		**0.00001**
**Ungulate diversity −**	−	27	0.47		**0.01**

^1^ Up to 2017: decline due to pneumonia epidemic which devastated the bighorn population. ^2^ Up until management reduced abundance after 2007.

**Table 4:** Grasshopper demographic and community responses to temperature and food factors based on best subset mixed model GLMM (site as random factor).

Grasshopper Measure	Impact	df
Hatch date (Julian day)		
June temperature	−, *p* < 0.00001	1,83
% Grass	−, *p* < 0.00001	1,83
r = 0.60, N = 90, AIC = 605.3		
Half-life (days)		
% Grass	−, *p* < 0.0001	1,119
Summer temperature	−, *p* < 0.007	1,119
June temperature	+, *p* < 0.05	1,119
r = 0.49, N = 123, AIC = 982.4		
Adult lifespan (days)		
June temperature	+, *p* < 0.006	1,85
% Grass	+, *p* < 0.01	1,85
r = 0.57, N = 93, AIC = 672.5		
Spring community (%)		
% Remain	+, *p* < 0.007	1,114
June temperature	+, *p* < 0.14	1,114
r = 0.34, N = 120, AIC = −551.3		
% *M. sanguinipes*		
% Grass	+, *p* < 0.00002	1,119
r = 0.75, N = 127, AIC = −104.3		
% *M. femur-rubrum*		
% Grass	−, *p* < 0.005	1,119
r = 0.70, N = 127, AIC = −167.5		
% *A. deorum*		
% Grass solubility	+, *p* < 0.002	1,113
% Grass	+, *p* < 0.005	1,113
June temperature	−, *p* < 0.12	1,113
r = 0.59, N = 115, AIC = −334.7		
Species richness		
% Dicot solubility	+, *p* < 0.002	1,107
% Grass	−, *p* 0.01	1,107
r = 0.69, N = 113, AIC = 402.65		
Species diversity		
% Grass	−, *p* < 0.01	1,118
June temperature	−, *p* < 0.03	1,118
r = 0.97, N = 127, AIC = 45.46		

**Table 5:** Population models (see text) fit using multiple stepwise regression along with statistics and AIC values.

Herbivore	1. Population	2. Food	3. Other	Combination
**Grasshoppers** **Avg. density**		grass_t−1_, + *p* < 0.11, N = 33 AIC = 189.3	Summer temp., − *p* < 0.006, N = 39 AIC = 219.8	2. and 3., (r = 0.53) N = 33 AIC = 183.2
**Microtines**	− *p* < 0.03, N = 30 AIC = 18.4	grass_t−1_, + *p* < 0.007, N = 29 AIC = 16.6		1. and 2. (r = 0.53) N = 25 AIC = 14.3
**Pronghorn**	+ *p* < 0.01, N = 34 AIC = 306.5	dicot_t−1_, + *p* < 0.07, N = 27 AIC = 250.5		1. and 2., (r = 0.59) N = 26 AIC = 236.0
**Deer**	+ *p* < 0.05, N = 24 AIC = 279.3	dicot_t−1_, + *p* < 0.19, N = 18 AIC = 209.0		1. and 2., (r = 0.48) N = 16 AIC = 186.8
**Bighorn**	+ *p* < 0.0001, N = 38 AIC = 306.0	grass_t−1_, + *p* < 0.0001, N = 30 AIC = 296.7		1. and 2., (r = 0.97) N = 30 AIC = 234.1
**Elk**	+ *p* < 0.003, N = 33 AIC = 335.1	grass_t−1_, + *p* < 0.08, N = 28 AIC = 293.8		1. and 2., (r = 0.51) N = 25 AIC = 259.0
Bison *	+ *p* < 0.04, N = 39 AIC = 401.6	grass_t−1_, + *p* < 0.03, N = 21 AIC = 223.9	remain_t−1_, + *p* < 0.01, N= 29 AIC = 229.3	1., 2. and 3., (r = 0.74) N = 21 AIC = 215.2

* NB bison from 1978 to 2007 due to management change after 2007 (see text).

## Data Availability

The original contributions presented in the study are included in the article/Supplementary Materials; further inquiries can be directed to the corresponding author.

## Supplementary Materials

The following supporting information can be downloaded at https://www.mdpi.com/article/10.3390/ani14182647/s1. Supplemental Table S1: The BR data for 1978–2019.
